# Individual Oral Therapy with Immediate Release and Effervescent Formulations Delivered by the Solid Dosage Pen 

**DOI:** 10.3390/jpm2040217

**Published:** 2012-11-06

**Authors:** Klaus Wening, Eva Julia Laukamp, Markus Thommes, Jörg Breitkreutz

**Affiliations:** Institute of Pharmaceutics and Biopharmaceutics, Heinrich-Heine-University, Duesseldorf 40225, Germany; E-Mails: klaus.wening@uni-duesseldorf.de (K.W.); julia.laukamp@uni-duesseldorf.de (E.J.L.); markus.thommes@uni-duesseldorf.de (M.T.)

**Keywords:** personalized medicine, individual therapy, delivery device, extrusion, pediatrics, geriatrics

## Abstract

New devices enabling freely selectable dosing of solid oral medications are urgently needed for personalized medicine. One approach is the use of the recently published Solid Dosage Pen, allowing flexible dosing of tablet-like sustained release slices from drug loaded extruded strands. Slices were suitable for oral single dosed application. The aim of the present study was the development of immediate release dosage forms for applications of the device, especially for young children. Using two model drugs, two different concepts were investigated and evaluated. Effervescent formulations were manufactured by an organic wet-extrusion process and immediate release formulations by a melt-extrusion process. Dissolution experiments were performed for both formulations to ensure the immediate release behavior. Extruded strands were individually dosed by the Solid Dosage Pen. Various doses of the two formulations were analyzed regarding uniformity of mass and content according to pharmacopoeial specifications. Proof of concept was demonstrated in both approaches as results comply with the regulatory requirements. Furthermore, storing stress tests were performed and drug formulations were characterized after storing. The results show that suitable packaging material has been selected and storage stability is probable.

## 1. Introduction

Individual dosing can be particularly important for highly potent drugs such as coumarin derivatives [1], personalized medicine [2,3,4] and the specific needs of younger or elderly patients [5,6,7,8]. The need for flexible dosing of solid dosage forms has been analyzed and currently available systems and development approaches have been reviewed [9]. Special demands of solid oral dosage forms for children with regard to formulations, excipients and acceptance issues have been discussed [10]. Moreover, delivery devices for the administration of pediatric formulations were recently reviewed and discussed by the European Paediatric Formulation Initiative (EuPFi) [11].

The opportunities for oral individual dosing so far include solid multiparticulate dosage forms like granulates, pellets or (mini-)tablets as well as liquid formulations like suspensions or solutions. Metering can be achieved via accumulation of multiple drug carriers or partition of monolithical forms [9]. Due to stability issues or drug solubility, liquid oral dosage forms are frequently limited [12]. Divisible tablets may inherit amongst others issues like difficulty of breaking, inaccuracy in dosing and loss of drug loaded mass [13]. However, with these dosage forms only a limited number of fixed compartments can be administered.

To individualize the dosing of an active pharmaceutical ingredient (API), innovative drug formulation concepts and novel devices or dispensing systems are needed. For those products, an appropriate design, a safe and easy handling for the patient, dose flexibility and expenditure have to be considered. Principle requirements in the field of pharmaceutics are dosage conformity and specified dissolution properties, which have to be independent of the delivered individual dose. Furthermore, chemical and physical stability of the dosage form over storage time and at different climate conditions has to be guaranteed.

A novel dosing device for individual therapy has recently been invented enabling the flexible dosing of a monolithical oral solid dosage form [14]. The developed dosing device has been named the Solid Dosage Pen, SDP [15]. Newly cylindrical solid dosage forms with sustained release characteristics have been developed and manufactured by a wet-extrusion process. The SDP can be used for the placement of these extrudates and dosage selection is carried out via an adjusting screw. Finally, individually dosed tablet-like slices can be cut off from the extrudates and be directly administered.

The aim of the present work was the development of immediate release formulations for the use of the SDP. These dosage forms should especially be suitable for pediatric medicines. Melt and wet-extrusion processes should be investigated for production of immediate release formulations. The dosing accuracy and stability of these novel formulations by using the SDP should be investigated.

As a first model drug carvedilol was chosen, which is slightly water-soluble. Its metabolism via CYP 2D6 is affected by gene variations. Further characteristics of the API which may require individual dosing are a therapeutic range over all age groups and daily doses for an adult only up to 20 mg.

Metoprolol tartrate as a second model substance shows high variability in pharmacokinetics due to metabolism of CYP 2D6, too [16]. Due to its high solubility in water and high therapeutic doses (200 mg daily standard dose for an adult) effervescent formulations might be favorable.

Both chosen model drugs—carvedilol and metoprolol tartrate—are administered as β-receptor antagonists in pediatric medicines. Cardiac insufficiency and heart failure are important applications [17] and so far no child-appropriate dosage form does exist [18].

## 2. Results and Discussion

### 2.1. Effervescent Formulation

#### 2.1.1. Formulation Development of Effervescent Formulation

Personalization in modern medicine requires new drug formulations. In this work as a first approach effervescent formulations of metoprolol tartrate for the use with the SDP were investigated for this purpose.

Today, dosing of effervescent formulations, especially for children, is only possible by dosing with dosing spoons or cups from bulk containers with powders or granules. This concept is unfavorable for dosing flexibility as well as for stability [9]. Once opened, the bulk container is susceptible for contamination and humidity. The graduation of dosing spoons for powdered material is quite difficult to read, therefore imprecise dosing is likely.

Furthermore, effervescent formulations belong to the most suitable formulations for children [19], especially for the therapy of very young children under five years. The idea of this feasibility study was to develop an effervescent formulation allowing the production of cylindrical dosage forms by a wet-extrusion process. These cylinders should be analyzed regarding their suitability for application via the SDP. The obtained doses should be evaluated for preparation of individually dosed drinkable solutions.

Extrusion is one common technique to obtain pharmaceutical cylindrical strands. The use of twin-screw extruders for effervescent formulations in general has been described by Lindberg *et al.* [20]. Based on hydrogen carbonate, citric acid and ethanol as liquid binder wet granules were produced. In the present study a die was applied at the extruder enabling a continuous production of drug-loaded rods. An adequate mechanical strength of the obtained rods after leaving the die was needed for conveying and further processing. Therefore a new composition had to be developed. In preliminary studies, an incompatibility was observed when using citric acid in combination with metoprolol tartrate. The observed strong fluidization of the formulation was avoided by using tartaric acid instead.

**Figure 1:**
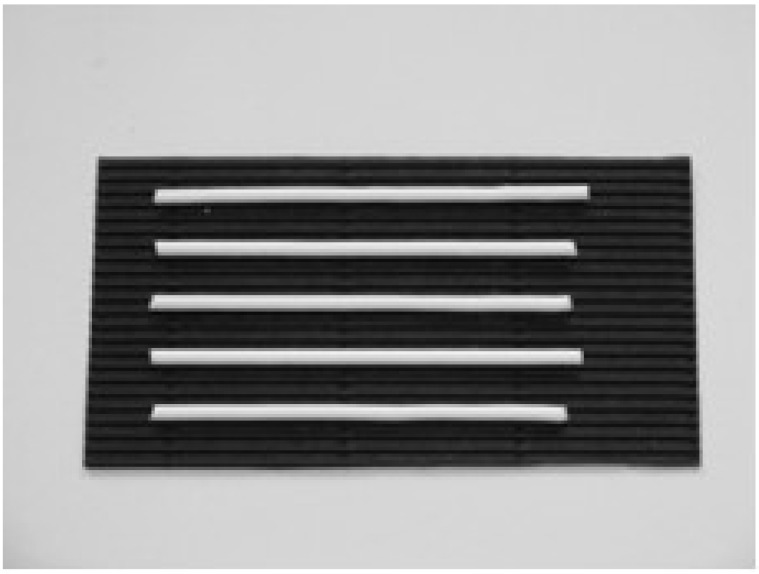
Dried effervescent strands (3 mm in diameter, approximately 5 cm length) obtained by twin-screw extrusion.

The powder mixture (Table 1) contained hydrogen carbonate, mannitol and tartaric acid with metoprolol tartrate. This mixture was extruded applying ethanol as granulation liquid in a ratio of 10 g/min solute to 90 g/min powder mixture. Thereby obtained cylindrical strands had a mechanical stability to be transported via a band-conveyer and to be transferred on a corrugated sheet for drying. Strands, having a diameter of 3 mm after drying, were cut into a length of approximately 5 cm (Figure 1). Storing was performed after sealing in airtight aluminum sachets.

**Table 1:** Composition of the powder mixture for the manufacturing of the effervescent formulation by a wet-extrusion process (%).

Effervescent formulation	
Metoprolol tartrate	18
Mannitol	38
Hydrogen carbonate	38
Tartaric acid	6

#### 2.1.2. Dosing of Effervescent Dosage Forms

**Figure 2:**
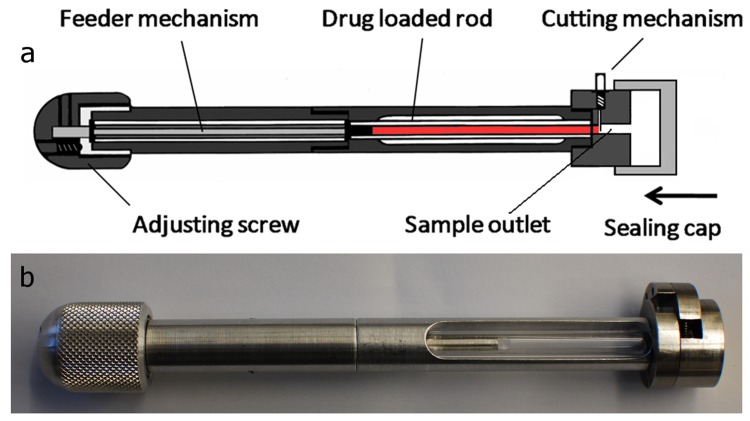
(**a**) Schematic drawing of modified SDP suitable for cutting of effervescent extrudates into individual doses; (**b**) Photograph of a constructed prototype.

For the application of the effervescent strands by the Solid Dosage Pen (SDP) a re-design of the device was required (Figure 2). In general, the working principle of the SDP is cutting off an individual selected dose from a drug loaded rod. Therefore the rod is located in the center of the device. By turning a screw-feed mechanism a defined feeding of the drug loaded strand was assured. The predefined part was then cut via an included cutting module. By pressing a mechanism outside of the module a cutting blade was pushed down to cut off a slice from the strand. The existing prototype of the SDP was only suitable for cutting soft and elastic strands. Brittle strands could not be cut by this design but were rather broken. To enable cutting of brittle strands from effervescent rods a modification of the previous cutting module of the SDP was made and a novel prototype was constructed consisting of several plastic and metal components (Figure 2).

This new design enabled cutting of the targeted dose from a brittle drug-loaded strand and its transfer through the sample outlet into water to prepare an oral solution. Even in case of obtaining multiple fragments when cutting the dose, the set-up ensures that all material will be transferred into the water for correct dosing. To avoid contacting ambient air, the device is additionally equipped with a sealing cap.

**Figure 3:**
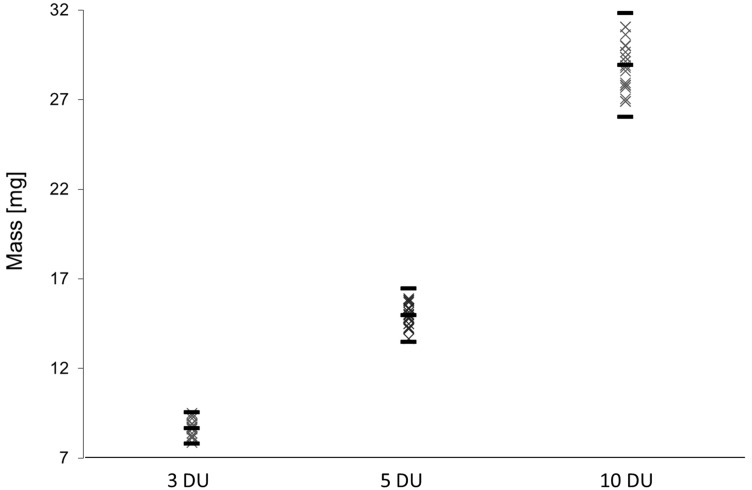
Masses of individually divided doses of the effervescent formulation using the SDP (d = 3.0 mm; 3, 5 und 10 dosage units, n = 20), arithmetic mean ± 10% according to Ph.Eur. 2.9.27.

Children, as well as elderly people suffering from disability or illness affecting manual strength, shall be able to utilize the SDP. To prove the working principle of the SDP with brittle strands, the forces needed to cut off doses were determined via force-displacement measurements. The resulting cutting force was 11.8 ± 1.2 N. These were considered to be comparably low forces if compared to other medical devices such as insulin pens or devices for pulmonary drug delivery and therefore no longer considered, as it is no critical issue for practical use [21]. In this context Kircher describes average manual forces for 20 to 60 year old healthy individuals between 55 N and 115 N, depending on the gender and on the pinch type (e.g., tip pinch, key pinch, chunk pinch) [21]. For individual dosing in pediatrics, dosing accuracy and uniformity of single doses is a challenge, due to low drug doses for children. For this purpose, the uniformity of dosing was analyzed by cutting off 20 doses for three different dosing regimens and determination of the uniformity of mass for these samples (Figure 3). 

Doses were differentiated by defining dosage units (DU). A dosage unit represents the unit which was exactly selected by the user of the SDP prototype, which equals in this case 0.5 mg metoprolol tartrate. As shown in Figure 3 the pharmacopoeial test “Uniformity of mass of single doses” was passed, as all single masses were within the specified 10% interval from arithmetic mean. Due to resulting doses of less than 25 mg the pharmacopoeial test “Uniformity of content of single doses” is also mandatory. Therefore, the API content of ten individual doses was determined. Results are shown in Table 2.

**Table 2:** Acceptance values according to Ph.Eur. 2.9.40 for effervescent single doses obtained by the SDP.

Dosage units (DU)	Nominal content of a single dose (mg)	Content (%)(MW ± SD)	Acceptance value(AV)
3	1.5	101.8 ± 4.5	11.18
5	2.5	101.4 ± 2.9	6.98
10	5	99.8 ± 3.1	7.49

Following European Pharmacopoeia, the resulting acceptance values have to be less than 15, which was fully accomplished in this work, even for low single doses. With an acceptable cutting force, a low mass variation and content uniformity the results proved the suitability of the SDP to cut individual doses from an effervescent solid rod. 

#### 2.1.3. Characterization of cut Effervescent Doses

**Figure 4:**
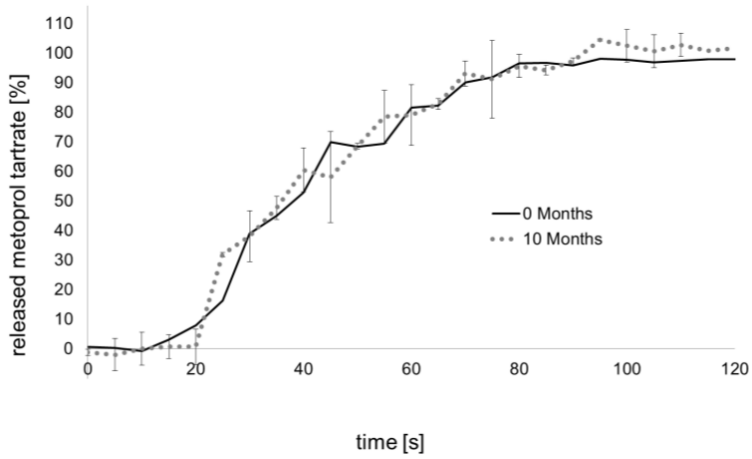
Dissolution of metoprolol tartrate from divided doses of the effervescent formulation (5 DU) determined via fiber optical probe before and after storing, 300 mL water, 20 °C, basket, 50 rpm, mean ± s (s: standard deviation), n = 3.

As disintegration of an immediate release dosage form and API dissolution are the first steps towards drug bioavailability, these properties of the cut doses were analyzed. The pharmacopoeia specifies that effervescent formulations have to disintegrate within five min in 200 mL of water at room temperature. Cut doses from rods with the novel formulation disintegrate in less than 5 min and hence, easily passed the test. The dissolution behavior of fast releasing dosage forms could not be determined by conventional dissolution tests. A modified method which has been used to analyze fast disintegrating orodispersible films [22] was used. The dissolution was performed in 300 mL of water at 20 °C. By using a fiber optical probe a spectrometically in-line measurement directly in the dissolution medium was applied with a measuring interval of five seconds. Resulting dissolution profiles are displayed in Figure 4.

The dissolution of the effervescent formulation showed a complete release within 80 s. The released drug cannot be homogenously distributed in the medium within this short interval, which led to relatively high standard deviations. Still, complete dissolution was accomplished.

Additionally, the extruded effervescent strands were stored in sealed aluminum sachets for ten months under accelerated conditions (40 °C, 75% relative humidity). Subsequently, the dissolution tests were repeated (Figure 4). As no changes in the dissolution profile were observed, it was concluded that drug release was guaranteed even after storage at accelerated conditions for ten months and the packaging material was suitable to preserve the effervescent strands. For the SDP a system has to be integrated which prevents the dosage form from humidity of the ambient air inside the device. This could be realized by using a drying material within the closure cap of the device.

In conclusion, the developed extruded effervescent formulation was dosed individually with the SDP to obtain precisely low dosed oral solutions, suitable especially for young children.

### 2.2. Immediate Release Melt-Extrudates

#### 2.2.1. Formulation Development of Immediate Release Melt-Extrudates

To broaden the application platform of the SDP, another development of immediate release solid oral dosage forms was intended. A melt-extrusion process based on polymers with low melting ranges was evaluated for the manufacturing of solid extrudates, which should be cuttable into tablet-like slices by the SDP. Metoprolol tartrate and carvedilol were used as model substances.

Based on solid poloxamers, a formulation development was made using a powder mixture for extrusion without addition of liquid plasticizers. Poloxamers are block-co-polymers of polyethylene oxide (PEO) and polypropylene oxide (PPO). Due to their molecular structure (PEO-PPO-PEO) they show amphiphilic characteristics and are typically used for solubility enhancement which has already been shown for carvedilol [23]. Solid poloxamers as excipients with wax characteristics have recently been introduced for melt-extrusion by Thommes *et al.* [24]. 

The drug formulations were based on a mixture of poloxamer 188 with mannitol. Carvedilol content was fixed to 5% and metoprolol tartrate to 20%. Poloxamer 188 was used in a coarse powder quality and a micronized quality. Resulting formulations are shown in Table 3.

**Table 3:** Composition of melt-extrudate batches (%).

Batch acronyms	LuCarv	LumiCarv	LuMet	LumiMet
Carvedilol	5	5		
Metoprolol tartrate			20	20
Mannitol	45	45	34	34
Poloxamer 188	50		46	
Poloxamer 188 micro		50		46

**Figure 5:**
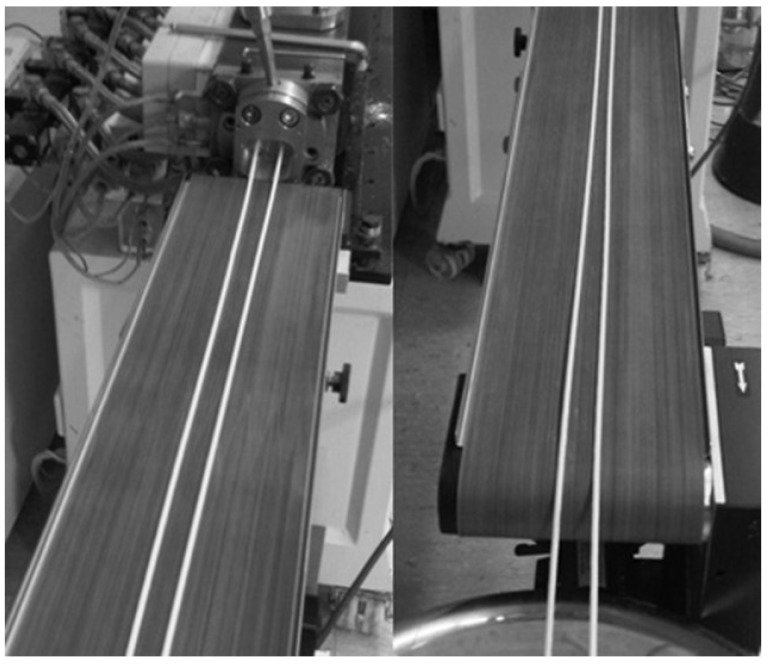
Cylindrical melt-extrudates (diameter 3 mm) based on Poloxamer 188 transported via a conveyer belt after extrusion to cool down.

All displayed drug formulations were extruded by applying a relatively low temperature of approximately 40 °C forming continuous extrudates with smooth shapes and a diameter of 3 mm. Extrudates were formed as straight drug-loaded rods and cooled down with the help of a conveyor belt as a downstream process (Figure 5). 

Finally, the strands were cut at a length of approximately 5 cm for the application with the SDP and stored in airtight aluminum sachets. 

#### 2.2.2. Dosing of Immediate Release Melt-Extrudates

Cutting forces needed to cut the extrudates into slices were determined for the melt-extrudates as well. For all drug formulations the forces were below 10 N and considered to meet the patients’ abilities.

**Figure 6:**
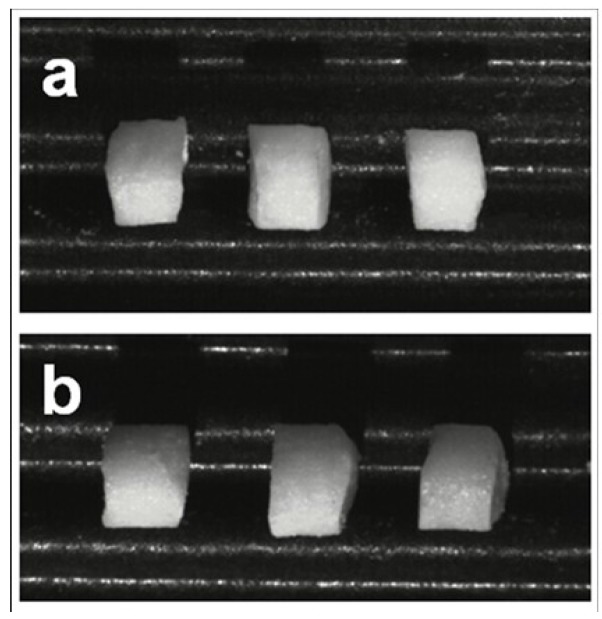
Single metoprolol doses divided by the SDP (d = 3 mm, 5 DU) of melt-extrudates with immediate-release characteristics. (**a**) LuMet; (**b**) LumiMet.

The drug loaded strands showed a slightly brittle behavior and were therefore applied by the redesigned SDP as shown in Figure 2. However, the targeted usage of the divided doses as single oral dosage forms made it necessary to obtain individual, exactly cut slices. Exemplarily cut doses from two formulations are shown in Figure 6.

The cut slices represent single doses; however, the optical appearance was worse compared to the results of sustained release formulations obtained by wet-extrusion process [15]. Further optimization should be made by using additional plasticizer to soften the strands for more accurate cutting and improved optical appearance. To analyze the uniformity of divided doses by the SDP drug loaded rods were placed into the device and 20 single doses were cut off. The uniformity of mass of the divided doses was determined. Despite the optical appearance the requirements of the European pharmacopoeia were met by all formulations with single doses consisting of five dosage units. All single masses were within the specified ranges. A dosage unit of the formulations with metoprolol tartrate contained approximately 0.75 mg and of formulations with carvedilol 0.2 mg API, respectively. Due to the resulting amount of less than 25 mg per single dose the acceptance value was determined exemplarily for two formulations resulting in a value of 7.5 for LumiMet and 8.7 for LumiCarv. Both values are clearly below the required threshold of 15. Therefore, the suitability of the formulations for dosing with the SDP has been demonstrated.

#### 2.2.3. Characterization of Cut Immediate Release Melt-Extrudate Doses

**Figure 7:**
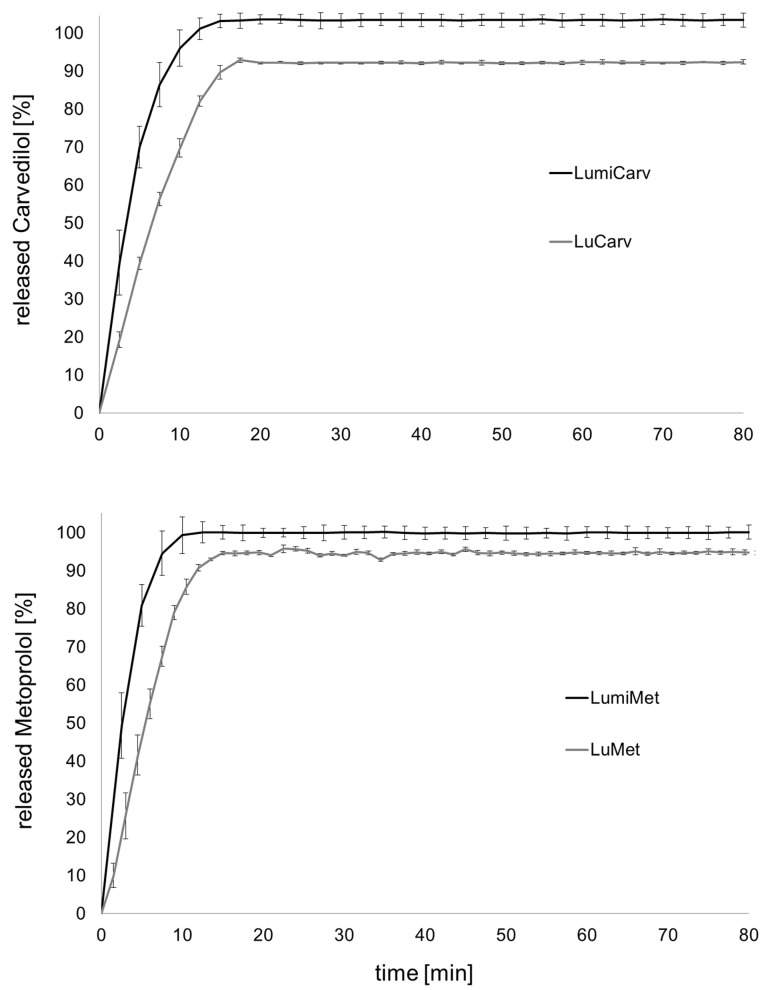
Dissolution profiles of divided doses from melt-extrudates, basket apparatus, 37 °C, 50 rpm, mean ± s, n = 6, 900 mL (**a**) carvedilol, 0.1 N HCl; (**b**) metoprolol tartrate, purified water.

Immediate release oral dosage forms have to release more than 85% of the API within 30 to 45 min (US Pharmacopoeia). To prove the immediate release behavior of the novel melt-extruded dosage forms a dissolution test was performed. The results are shown in Figure 7. 

**Figure 8:**
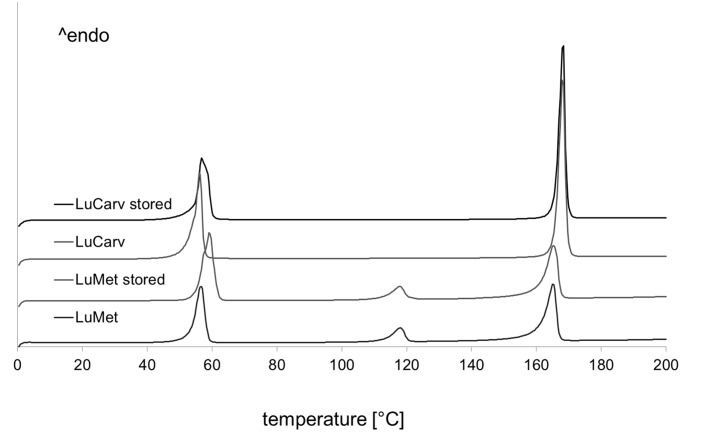
DSC thermograms of melt-extrudates; LuCarv und LuMet before and after storing for nine months at accelerated conditions (40 °C, 75% relative humidity).

All analyzed formulations released the contained API dose within 20 min. Formulations containing the standard grade of Poloxamer 188 released less than 100% of the API, which was overcome by usage of the micronized Poloxamer 188 grade. Insufficient powder blending due to various particle sizes of the ingredients or segregation of the powder mixture might be an explanation for this observation. It is recommended by the authors to use the micronized grade of poloxamer 188 for powder blending. Alternatively, split-feeding could overcome this challenge. The release of carvedilol was surprisingly fast as the solubility is quite low (1 mg/mL at pH 1). It can be concluded that the poloxamer had a positive influence on the dissolution of carvedilol. A differential scanning calorimetry (DSC) analysis of the extrudates was performed to analyze the solid state behavior of the formulations LuCarv and LuMet (Figure 8). In the DSC thermograms the melting points of the excipients could clearly be detected at 55 °C and 166 °C for poloxamer and mannitol, respectively. The two APIs showed a discriminative behavior in the thermograms: A melting endotherm of carvedilol could not be detected whereas the melting endotherm of metoprolol tartrate was clearly detectable at 118 °C. 

This behavior was supported by further DSC measurements with various powder mixtures and additionally via X-ray powder diffractometry (data not shown). We conclude that carvedilol was completely dissolved in the poloxamer matrix resulting in fast dissolution. Investigations of powder mixtures containing mannitol and poloxamer with increasing amount of carvedilol showed that only carvedilol concentrations of more than 30% show an endothermic peak of carvedilol in DSC analysis (data not shown).

DSC analysis test was also applied for the extrudates after stressing at accelerated conditions in aluminum sachets for nine months, as displayed in Figure 8. For both formulations no changes were observed for the included active pharmaceutical ingredient. Therefore, a physical stability of the drug-loaded strands can be anticipated.

**Figure 9:**
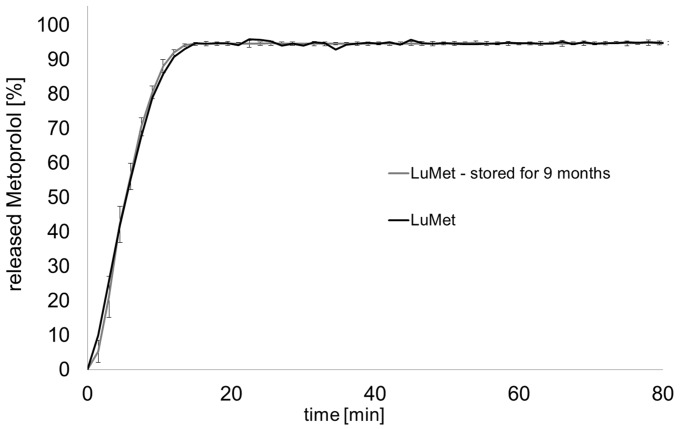
Dissolution of cut slices from metoprolol tartrate extrudates by the SDP (d = 3 mm) before and after storing nine months at accelerated conditions, 900 mL water, 37 °C, basket apparatus, 50 rpm, mean ± s, n = 6.

Exemplarily, the dissolution profile of one formulation after storing for nine months at accelerated conditions is displayed in Figure 9 showing no changes in the dissolution profile for the formulation LuMet. 

Stability testing indicates that aluminum sachets might be a suitable packaging material to protect the drug loaded strands from ambient moisture.

Summarizing the results, melt-extrusion is a promising approach for the production of immediate release strands for application with the SDP. Further investigations shell be implemented to optimize the formulations regarding appearance of the divided doses.

## 3. Experimental

### 3.1. Materials

Sodium hydrogen carbonate (Emprove^®^, Merck, Darmstadt, Germany), mannitol (Pearlitol^®^ 160C, Roquette, Lestrem, France), tartaric acid (Caesar & Loretz, Hilden, Germany), and poloxamer 188 (Lutrol^®^ F68micro and Lutrol^®^ F68, BASF, Ludwigshafen, Germany) were used as received. Metoprolol tartrate (Microsin, Bucharest, Romania) and carvedilol (Salutas Pharma, Barleben, Germany) were used as model drugs. 

### 3.2. Methods

#### 3.2.1. Extrusion

A co-rotating twin-screw extruder Micro 27GL/28D (Leistritz, Nuremberg, Germany) equipped with three-hole die plate with 2.7 mm in diameter and 0.5 mm in length was used. Powder components were blended for 15 min in a laboratory scale blender (LM 40, Bohle, Ennigerloh, Germany) and afterwards transferred into a gravimetric feeder (K-CL-KT 20, K-Tron Soder, Niederlenz, Switzerland). For wet-extrusion ethanol 96% was used as liquid binder supplied by a membrane pump (Cerex EP-31, Bran and Luebbe, Norderstedt, Germany) with a flow through metering device (Corimass MFC-081/K, Krohne, Duisburg, Germany). Temperature for wet-extrusion was applied to 25 °C and for melt-extrusion at 40 °C, screw rotation speed was adapted for each process. A conveyer belt (Brabender, Duisburg, Germany) with adaptable speed was used to obtain straight extruded strands. Wet-extrudates were transferred on a corrugated well sheet and dried during 60 °C for 30 min.

#### 3.2.2. Drug Release

**Figure 10:**
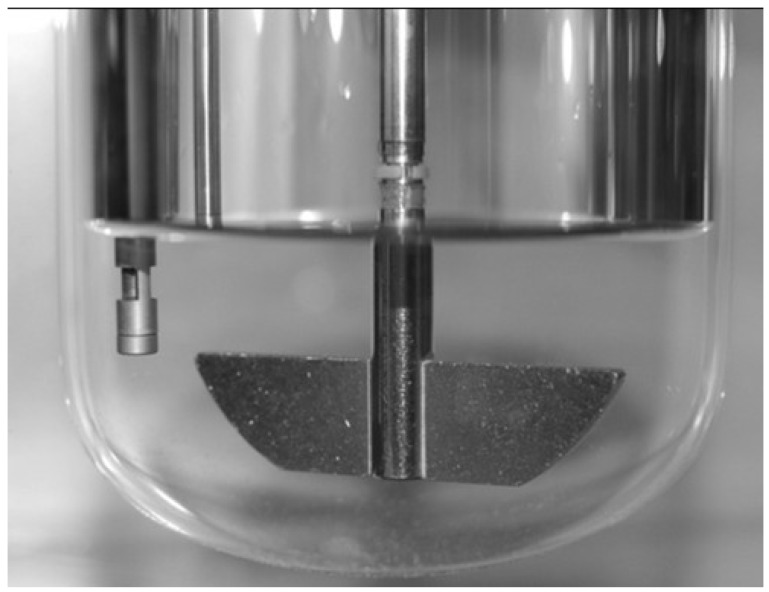
Modified dissolution setup to perform in-line dissolution monitoring of rapidly releasing drug dosage forms using paddle apparatus and 300 mL dissolution fluid.

Dissolution tests according to Ph.Eur. Method 2.9.3 was performed with melt-extrudates using paddle apparatus Sotax AT 7 (Sotax, Lörrach, Germany). Experiments were conducted in 900 mL 0.1-N hydrochloric acid or purified water at a stirring speed of 50 rpm and a temperature of 37 ± 0.5 °C. The concentration of released drug was determined using a UV-spectrometer Lambda 40 (Perkin Elmer, Überlingen, Germany). Effervescent formulations were analyzed using a modified setup (Figure 10) including a fiber optical system (Micropack, Ostfildern, Germany). The system consists of a DH-2000-BAL deuterium tungsten halogen light source, a USB-4000-UV-VIS optical bench and a T300-RT transmission dip probe for in-line monitoring. Components are connected by optical fibers containing an illumination and a read fiber. The light is transmitted from the illumination fiber through a plano-convex lens through the media onto a flat mirror (distance 0.5 mm), from which the light is reflected and focused by the lens to the read fiber. The signals are determined by the optical bench and transformed for evaluation by Spectra Suite^®^ software.

#### 3.2.3. Uniformity of Content of Single Doses

The API content of ten doses each was determined from aqueous solution using an UV-spectrometer, Lambda 40 (Perkin Elmer, Überlingen, Germany) at the wavelengths 284 nm (carvedilol) and 223 nm (metoprolol tartrate). The acceptance value according to the Ph. Eur. method 2.9.40 was determined.

#### 3.2.4. AV-Value

The acceptance value according to the method 2.9.40 from the European Pharmacopeia was calculated. Therefore the API content of ten doses each was determined from aqueous solution using an UV-spectrometer, Lambda 40 (Perkin Elmer, Überlingen, Germany). A suitable wavelength was applied. The acceptance value (AV) was calculated by Equation (1).


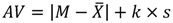
(1)

#### 3.2.5. Differential Scanning Calorimetry (DSC)

The thermal properties of the extrudates were analyzed using a DSC-1 (Mettler-Toledo, Giessen, Germany). Approximately 5 mg of samples were sealed in a pierced aluminum pans and an empty pan was used as reference. The heating rate was 10 g/min from 0 °C to 300 °C. The chamber was flooded with nitrogen. Data collection and treatment was made by Star^e^ 9.20 software (Mettler-Toledo). All measurements were performed in duplicate.

#### 3.2.6. Determination of Cutting-Forces

Cutting forces, defined as maximum force for successful cutting, were determined by Test Apparatus H10KM (Hess, Sonsbeck, Germany) equipped with a 1,000 N load cell. A specifically developed cutting tool [15] was used applying a cutting speed of 100 mm/min. Force-displacement tracks of 20 samples each were recorded and maximum forces during cutting process were determined.

## 4. Conclusions

The aim of this feasibility study was to develop immediate release formulations for application via the Solid Dosage Pen as a novel device for treatment in personalized medicine. Two different approaches were investigated to obtain drug loaded strands via an extrusion processes. First, cylindrical effervescent formulations were manufactured by a wet-extrusion process. Second, a new immediate release formulation was developed for manufacturing strands by a melt-extrusion process. The investigations of the cuttability, the maximum cutting forces and the uniformity of the cut doses demonstrated that both types of drug formulations were applicable with the SDP to deliver freely selectable individual doses. The obtained doses were characterized concerning immediate release behavior as well as physical and chemical stability. Feasibility was proven for both concepts with slightly water-soluble carvedilol and freely soluble metoprolol tartrate as model substances. Therefore, the recently introduced Solid Dosage Pen is a device which was found to be applicable to an oral individual therapy for various target groups and with a broad range of application variations, perfectly matching the approaches of personalized medicine.
